# Hemoglobin stability impact on healthcare resource utilization and costs among dialysis-dependent patients with anemia of end-stage kidney disease

**DOI:** 10.1186/s12882-025-04390-y

**Published:** 2025-08-18

**Authors:** Anna D. Coutinho, Malena Mahendran, Maral DerSarkissian, Sophie A. Kitchen, Christopher F. Bell, Mary Muoneke, Anna Richards

**Affiliations:** 1 GSK, 1250 S Collegeville Rd, Collegeville, PA 19426 USA; 2 Analysis Group, Washington DC, USA; 3 Analysis Group, Los Angeles, CA USA; 4 GSK, Durham, CA USA; 5 GSK, London, UK

**Keywords:** End-stage kidney disease, Dialysis-dependent, Hemoglobin stability, Healthcare resource utilization, Red blood cell transfusion, Time within hemoglobin target range, Optum^®^ market clarity

## Abstract

**Background:**

The impact of hemoglobin stability on healthcare resource utilization (HCRU) and costs in dialysis-dependent patients with anemia of end-stage kidney disease (ESKD) was evaluated.

**Methods:**

This retrospective, observational study used Optum’s de-identified Market Clarity Data (Optum^®^ Market Clarity) (2017–2019). Dialysis-dependent patients with anemia of ESKD were stratified by mean hemoglobin level (below, within, or above the target range of 10.0–11.5 g/dL) and time within hemoglobin target range (TiR; high, ≥ 60% or low, < 60% of hemoglobin measurements within the target range) over a 9-month exposure assessment period following initiation of anemia treatment. Incidence rates of HCRU per-person year and annual mean costs were compared between groups weighted by inverse probability weighting during the outcomes assessment period (day after exposure assessment period until earliest of renal transplantation date, end of eligibility, or data availability [March 31, 2022], or death).

**Results:**

Of 2,279 eligible patients, 37.1%, 44.1%, and 18.8% had mean hemoglobin levels below, within, and above the target range, respectively, and 78.2% had low TiR. Patients with hemoglobin levels within the target range had a significant cost reduction of $6,201 in red blood cell (RBC) transfusions, and significantly lower incidence of RBC transfusions (46%) and inpatient visits (20%) compared to those below the target range. Mean hemoglobin level within versus above the target range was associated with a significantly higher incidence rate of inpatient visits (14%) and annual costs ($1,958) for RBC transfusions. Patients with high versus low TiR had significantly lower incidence rate of RBC transfusion visits (43%) and inpatient visits (19%), and a significant total healthcare cost reduction of $33,921.

**Conclusions:**

Increasing hemoglobin levels to within the target range, and having a higher TiR, was associated with positive impacts on RBC transfusion frequency, HCRU, and costs among dialysis-dependent patients with anemia of ESKD.

**Clinical trial number:**

Not applicable.

**Supplementary information:**

The online version contains supplementary material available at 10.1186/s12882-025-04390-y.

## Background

Anemia is a frequent complication of end-stage kidney disease (ESKD) [1], defined as a hemoglobin level < 12 g/dL for females and < 13 g/dL for males [2, 3]. Treatment options for patients with anemia of ESKD include erythropoiesis-stimulating agents (ESAs), iron supplements (intravenous [IV] or oral), hypoxia inducible factor prolyl hydroxylase inhibitors, and, if indicated, red blood cell (RBC) transfusions [3–7]. Managing dialysis-dependent patients with anemia of ESKD is challenging as hemoglobin levels fluctuate with ESAs and iron use [8, 9]. Over 90% of patients on chronic hemodialysis experience repeated hemoglobin fluctuations [8] which are associated with an increased risk of hospitalizations, morbidity, and mortality [10].

Prior to 2010, clinical practice guidelines recommended treating patients to a hemoglobin target range of 11.0–12.0 g/dL [11]. This target was updated to 10.0–11.5 g/dL in 2012 [3], based on pivotal ESA trials demonstrating an increased risk of major adverse cardiovascular events in patients with hemoglobin levels above 11.5 g/dL [12–16]. Managing anemia in dialysis-dependent patients is complicated by fluctuations in hemoglobin levels, the occurrence of which is well-documented in dialysis-dependent patients treated with ESAs and IV iron [8]. Several studies evaluating the impact of hemoglobin variability on hospitalization and mortality found that hemoglobin excursions above or below target range (and more often below) were associated with increased risk of hospitalization, length of hospitalization stay, and mortality [17–22].

Fluctuations in hemoglobin levels have been associated with increased healthcare resource utilization (HCRU) and costs [22–24]. Despite the clinical benefits of treating patients with anemia of ESKD who are dialysis-dependent, data on the economic impact of maintaining hemoglobin levels within the recommended target range (10.0–11.5 g/dL) are sparse. To elucidate the impact of hemoglobin stability on the economic burden of anemia of ESKD, this study assessed HCRU and costs in a US dialysis-dependent population primarily covered by Medicare Advantage or commercial health insurance stratified by mean hemoglobin levels and time-in-range (TiR).

## Methods

### Study design

**Fig. 1:**
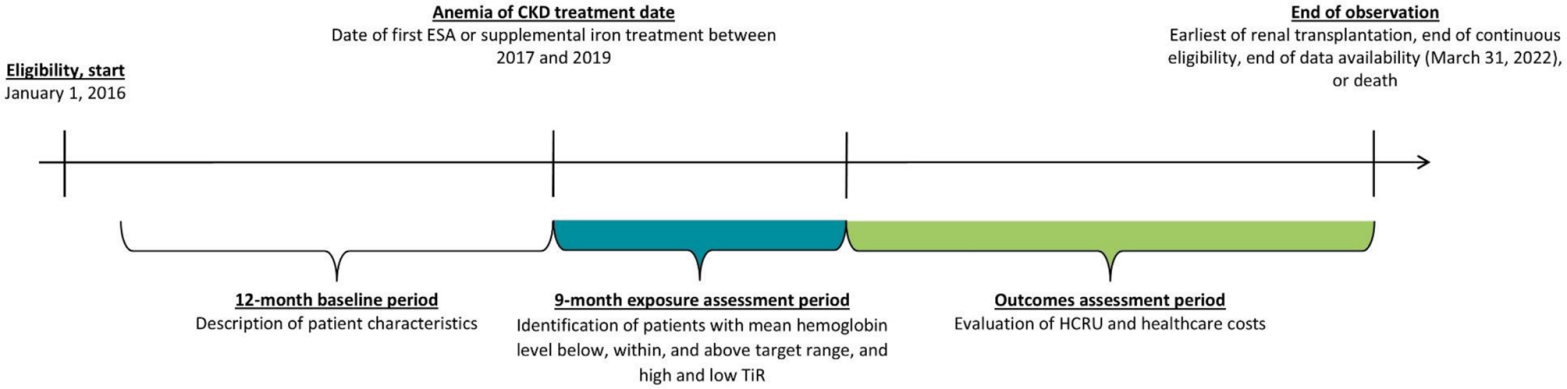
Study design. CKD, chronic kidney disease; ESA, erythropoiesis-stimulating agents; HCRU; healthcare resource utilization; TiR, time in range

This retrospective, observational study used de-identified medical and pharmacy claims linked to electronic health records from the Optum’s de-identified Market Clarity Data (Optum^®^ Market Clarity), from January 1, 2016 to March 31, 2022 (end of data availability) [25, 26]. The date of the first medical or pharmacy claim for ESA or supplemental iron, between January 1, 2017 and December 31, 2019, was defined as the anemia of ESKD treatment date (Fig. 1). Hemoglobin stability was assessed during a 9-month exposure assessment period following the anemia of ESKD treatment date to allow sufficient time to achieve hemoglobin stability as patients newly treated with ESA and iron supplementation would have their dose titrated 6–9 months following treatment initiation. Patient characteristics were described during the 12 months preceding the anemia of ESKD treatment date (baseline). All-cause HCRU and costs were quantified during the outcomes assessment period, spanning from the day after the end of the exposure assessment period to the earliest date of renal transplantation, end of eligibility, end of data availability, or death.

## Study population

Eligible patients with anemia of ESKD were aged ≥ 18 years and dependent on hemodialysis or peritoneal dialysis, defined as ≥ 1 medical or pharmacy claim for hemodialysis or peritoneal dialysis for ≥ 3 consecutive months during the baseline period and up to 4 months after the anemia of ESKD treatment date. Patients were required to have ≥ 1 prescription, dispensing, or administration of ESA or supplemental iron (IV or oral) between January 1, 2017 and December 31, 2019, continuous health insurance coverage through the baseline and exposure assessment periods, and ≥ 2 hemoglobin measurements during the exposure assessment period.

To exclude patients with non-ESKD-related causes of anemia, patients with ≥ 1 record of renal transplant or ≥ 1 diagnosis of active bleeding, hemolytic anemia, sickle cell anemia, aplastic anemia, anemia due to enzyme disorders, nutritional anemias, malignancies, myelofibrosis, or myelodysplasia on the anemia of ESKD treatment date or during the baseline period were excluded.

## Study comparator groups

During the 9-month exposure assessment period, patients were categorized into groups defined by two metrics of hemoglobin stability and compared as follows: (a) mean hemoglobin level below (< 10.0 g/dL) versus within the target range (10.0‒11.5 g/dL) and above (> 11.5 g/dL) versus within the target range; (b) high TiR (≥ 60% of hemoglobin measurements within 10.0‒11.5 g/dL) versus low TiR (< 60% of hemoglobin measurements within 10.0‒11.5 g/dL). The threshold of ≥ 60% for TiR was based on the findings of a recent trial of a novel treatment for anemia of chronic kidney disease (CKD) in patients who were undergoing dialysis [4, 27].

## Study outcomes

The primary aim of this study was to describe and compare all-cause HCRU among dialysis-dependent patients with ESKD who had a mean hemoglobin level below, within, or above the target range (10.0‒11.5 g/dL). Patterns of hemoglobin measurements during the exposure assessment period included mean hemoglobin level, distribution below the target range, number of measurements, and time between measurements. All-cause HCRU included hospitalizations, emergency room visits, and RBC transfusion visits. Incidence rates of HCRU per person year (PPY) were calculated as the number of events divided by total person-years of observation.

Secondary aims were to describe patient demographics and clinical characteristics by mean hemoglobin level below, within, or above the target range and TiR; describe patterns of hemoglobin level following initiation of anemia of CKD treatment; report all-cause HCRU and costs by hemoglobin level below, within, or above target range and among patients with high TiR (≥ 60% of all hemoglobin measurements in target range) versus low TiR (< 60% of all hemoglobin measurements in target range). All-cause costs analyzed across all years were inflation-adjusted to 2022 US dollars (i.e., the latest year of data available), based on the medical care component of the Consumer Price Index and included total healthcare costs (medical and pharmacy costs associated with the receipt of ESAs and supplemental iron ([IV or oral]), medical costs (hospitalization costs, emergency room [ER] visit costs, and RBC transfusion visit costs), and pharmacy costs. Costs per person per year (PPPY) were calculated at the patient-level by dividing the costs incurred over the outcomes assessment period by the number of person-years of observation and reported as mean with standard error.

### Sensitivity analysis

Outcomes between groups defined by mean hemoglobin level were assessed in patients newly treated and previously treated with ESA or supplemental iron. Newly treated patients were defined as those who did not receive ESA or supplemental iron treatment for anemia of ESKD during the baseline period. Previously treated patients were defined as those who received ESA or supplemental iron during the baseline period. For TiR, a sensitivity analysis varying the threshold from ≥ 60% to ≥ 50% was conducted in the overall population.

## Statistical analyses

Patient characteristics were summarized using descriptive statistics, including mean with standard deviation (SD) and median with interquartile range for continuous characteristics and frequencies with proportions for categorical characteristics. Differences in baseline characteristics between comparator groups were assessed using standardized differences, with a difference > 10% indicating a meaningful imbalance between groups. Clinically relevant characteristics and those with meaningful imbalances were included in the propensity score model to derive the weights for inverse probability weighting (IPW). IPW was used to minimize potential confounding and achieve optimal balance between comparator groups. Incidence rates of HCRU were compared between IPW-weighted comparator groups using incidence rate ratios (IRRs), 95% confidence intervals (CIs), and *P*-values estimated from negative binomial regression models with a zero-inflation parameter if excess zero values were observed. Mean costs were compared between IPW-weighted comparator groups using mean cost differences, 95% CIs, and *P*-values estimated from 2-part gamma regression models to account for excess zero values. For all comparative analyses, a two-tailed, type I error rate of 0.05 was the threshold for statistical significance. All analyses were conducted using SAS Enterprise Guide, Version 8.4 (SAS Institute, Cary, NC, USA).

## Ethics approval and consent to participate

This study complied with the ethical principles of the Declaration of Helsinki and applicable legislation on observational studies. The Optum’s de-identified Market Clarity Data (Optum^®^ Market Clarity) were de-identified and compliant with applicable privacy laws. No direct patient contact or primary collection of individual patient-level data occurred. Study results are presented as aggregate analyses that omit individual identification in compliance with the patient confidentiality requirements of the Health Insurance Portability and Accountability Act (HIPAA) 1996. Therefore, this study did not require informed consent or review/approval from an ethics committee or institutional review board.

## Results

### Patient characteristics

**Fig. 2:**
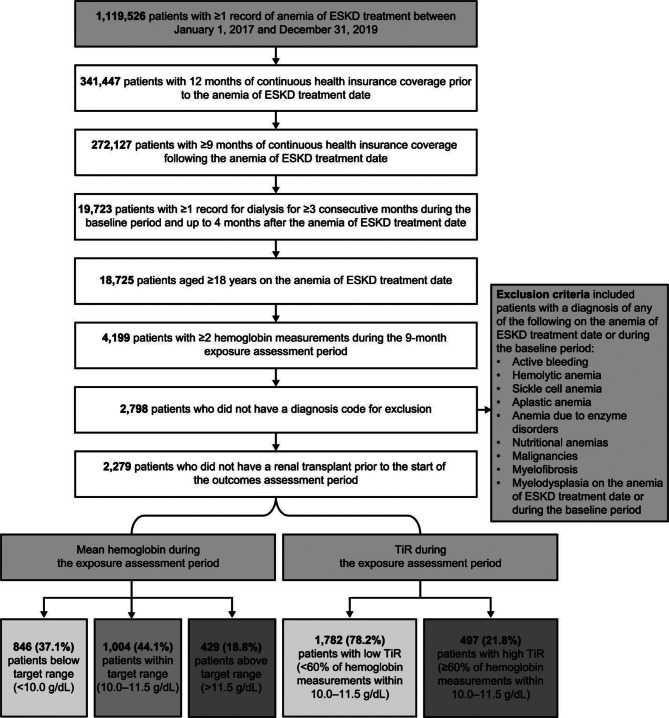
Patient selection from eligibility criteria. ESKD, end-stage kidney disease; TiR, time-in-range

A total of 2,279 patients met al.l study criteria (Fig. 2). During the exposure assessment period, less than half (44.1%) of patients had a mean hemoglobin level within the target range, 37.1% were below the target range, and 18.8% were above the target range. The majority (78.2%) had low TiR.

Across the unweighted patient characteristics of comparator groups defined by mean hemoglobin level and TiR, Asian race and ethnicity had standardized differences > 10% (Supplementary Table 1). ESA treatment and all dialysis modalities differed meaningfully between groups defined by mean hemoglobin level, while dialysis vintage was longer in patients with high TiR.

After weighting, patient demographics and clinical characteristics were well balanced across all groups, except for ethnicity (Table 1). Mean age was 61.4–61.7 years; approximately half were male (53.7–54.3%). Most patients received both ESA and supplemental iron (78.1–79.4%), hemodialysis (85.0–87.3%), and dialysis for longer than 4 months (65.5–66.8%). Common ESKD-related comorbidities were present among all patient groups including, hypertension (95.2–97.2%), diabetes (71.0–73.4%), hyperlipidemia (68.1–72.7%), and heart failure (55.6–57.3%). Over half (56.6%) of patients were newly treated with ESA or supplemental iron. Like the overall population, less than half of the patients in the newly (41.1%) and previously treated (47.9%) subgroups had mean hemoglobin levels within the target range.

**Table 1:** Patient characteristics by study comparator groups (weighted^a^ sample)

	**Mean hemoglobin during the exposure assessment period**^**b**^				**TiR during the exposure assessment period**^**c**^		
Characteristic	Below target range (N = 846)	Within target range (N = 1,004)	Above target range (N = 429)	Std. diff (%)	Low TiR (N = 1,782)	High TiR (N = 497)	Std. diff (%)
**Age in years, mean (SD)**	61.5 ± 13.3	61.4 ± 13.3	61.7 ± 12.6	−0.58	61.4 ± 13.2	61.6 ± 12.9	1.55
**Age in years, median (IQR)**	63 (54,72)	63 (53,72)	62 (54,71)	─	63 (54,72)	63 (53,71)	─
**Male, *n (%)***	454 (53.7)	541 (53.9)	232 (54.2)	0.39	960 (53.9)	270 (54.3)	0.75
**Race, *n (%)***							
White	433 (51.2)	509 (50.7)	219 (51.0)	−0.96	897 (50.4)	254 (51.1)	1.58
African American/Black	309 (36.5)	372 (37.1)	158 (36.8)	1.23	670 (37.6)	183 (36.9)	−1.43
Asian	18 (2.2)	22 (2.1)	8 (1.8)	−0.05	38 (2.1)	11 (2.2)	0.68
Other/unknown	86 (10.2)	101 (10.1)	45 (10.4)	−0.35	177 (10.0)	48(9.8)	−0.66
**Ethnicity, *n (%)***							
Not Hispanic/Latino	705 (83.3)	792 (78.8)	341 (79.5)	**-11.53†**	1,461 (82.0)	383 (77.0)	**-12.46†**
Hispanic/Latino	66 (7.8)	125 (12.5)	48 (11.3)	**15.43†**	167 (9.4)	64 (12.9)	**11.13†**
Unknown	74 (8.8)	87 (8.7)	39 (9.2)	−0.51	153 (8.6)	50 (10.1)	5.23
**Medical insurance type, *n (%)***							
Medicare	425 (50.3)	500 (49.8)	214 (49.9)	−0.93	891 (50.0)	250 (50.3)	0.57
Commercial	230 (27.2)	276 (27.5)	114 (26.6)	0.67	489 (27.5)	137 (27.5)	0.11
Medicaid	127 (15.0)	152 (15.2)	66 (15.4)	0.41	267 (15.0)	73 (14.7)	−0.79
Unknown	64 (7.5)	76 (7.5)	35 (8.1)	0.08	135 (7.6)	37 (7.5)	−0.20
**Anemia of ESKD treatment during the exposure assessment period, *n (%)***							
ESA only	89 (10.6)	104 (10.3)	40 (9.3)	−0.78	186 (10.4)	52 (10.4)	−0.03
Supplemental iron only	95 (11.2)	115 (11.5)	49 (11.3)	0.75	203 (11.4)	57 (11.5)	0.20
Both	662 (78.2)	785 (78.2)	341 (79.4)	0	1,393 (78.2)	388 (78.1)	−0.13
**Dialysis treatment modalities, *n (%)***							
Hemodialysis	720 (85.1)	854 (85.0)	375 (87.3)	−0.03	1,517 (85.1)	425 (85.5)	1.00
Peritoneal dialysis	112 (13.2)	128 (12.7)	51 (11.8)	−1.50	231 (12.9)	64 (13.0)	0.02
Unknown	85 (10.1)	101 (10.1)	37 (8.6)	0.16	178 (10.0)	48 (9.7)	−1.07
**Dialysis vintage, *n (%)***							
1–4 months	287 (34.0)	342 (34.1)	148 (34.5)	0.29	601 (33.7)	165 (33.2)	−1.05
> 4 months	559 (66.0)	662 (65.9)	281 (65.5)	─	1,181 (66.3)	332 (66.8)	─
**Dialysis vintage, months**							
Mean (SD)	7.4 (5.0)	7.3 (5.0)	7.3 (4.9)	−1.28	7.4 (5.0)	7.5 (4.9)	1.66
Median (IQR)	11 (1, 12)	10 (1, 12)	10 (1, 12)	─	10 (1, 12)	10 (2, 12)	─
**ESKD-related comorbidities, *n (%)***							
Hypertension	805 (95.2)	957 (95.3)	417 (97.2)	0.67	1,703 (95.6)	474 (95.4)	−0.98
Diabetes	618 (73.1)	713 (71.0)	315 (73.4)	−4.62	1,287 (72.2)	361 (72.5)	0.78
Hyperlipidemia	576 (68.1)	710 (70.7)	311 (72.4)	5.56	1,230 (69.0)	361 (72.7)	7.98
Heart failure	476 (56.2)	560 (55.8)	239 (55.7)	−0.88	992 (55.6)	285 (57.3)	3.44

†Standardized difference > 10% indicative of a meaningful imbalance between groups

^a^Weights were derived from the inverse of the propensity score generated from multinomial regression models adjusting for potential confounding variables. The following variables were included in the propensity score models: age, sex, race, geographic region, medical insurance type, year of anemia ESKD treatment, type of anemia ESKD treatment over the exposure assessment period, dialysis treatment modalities, dialysis vintage, proportion of patients switching dialysis treatment modalities, other cardiovascular disorder, arteriosclerotic heart disease, chronic obstructive pulmonary disorder, Quan-Charlson comorbidity index, BMI, and all-cause HCRU

^b^Patients were classified as having a mean hemoglobin level below the target range (< 10.0 g/dL), within the target range (10.0‒11.5 g/dL), or above the target range (> 11.5 g/dL)

^c^Patients were classified as having either a high time-in-range (TiR; ≥ 60% of all hemoglobin measurements within the target range of 10.0‒11.5 g/dL) or a low TiR (< 60% of all hemoglobin measurements within the target range of 10.0‒11.5 g/dL)

BMI, body mass index; ESA, erythropoiesis-stimulating agent; ESKD, end-stage kidney disease; HCRU, healthcare resource utilisation; IQR, interquartile range; SD, standard deviation; Std. diff., standardized difference; TiR, time-in-range

## Hemoglobin levels during the exposure assessment period

In the overall population, the mean (SD) hemoglobin level was 10.4 (1.3) g/dL during the exposure assessment period (Table 2). Among patients with a mean hemoglobin level below the target range, more than half (59.9%) had a hemoglobin level between 9.0–9.9 g/dL. This group also had the shortest time between hemoglobin measurements (1.4 months) compared to 1.8 and 2.1 months for the within and above the target range groups. Among patients with a mean hemoglobin level above the target range, the mean hemoglobin level was 12.3 (0.8) g/dL with 75% having a value of < 12.6 g/dL. This group also had the lowest mean number of hemoglobin measurements (4.2) compared to patients with mean hemoglobin level within (5.9) or below (5.8) the target range over the exposure assessment period. Mean (SD) hemoglobin levels were similar for patients in the low (10.3 [1.5] g/dL) and high (10.7 [0.5] g/dL) TiR groups, as were number of and average time between hemoglobin measurements.

**Table 2:** Hemoglobin levels and measurements during the exposure assessment period by study comparator groups (unweighted sample)

	All patients (N = 2,279)	**Mean hemoglobin during the exposure assessment period**^**a**^			**TiR during the exposure assessment period**^**b**^	
		Below target range (N = 846)	Within target range (N = 1,004)	Above target range (N = 429)	Low TiR (N = 1,782)	High TiR (N = 497)
**Hemoglobin level (g/dL)**						
Mean (SD)	10.4 (1.3)	9.1 (0.7)	10.7 (0.4)	12.3 (0.8)	10.3 (1.5)	10.7 (0.5)
Median (IQR)	10.4 (9.5, 11.2)	9.2 (8.6, 9.7)	10.7 (10.4, 11.1)	12.1 (11.7, 12.6)	10.1 (9.3, 11.4)	10.8 (10.5, 11.1)
**Hemoglobin categories below target range, *n (%)***						
< 8.0 g/dL	76 (3.3)	76 (9.0)	0	0	76 (4.3)	0
8.0‒8.9 g/dL	263 (11.5)	263 (31.1)	0	0	263 (14.8)	0
9.0‒9.9 g/dL	507 (22.2)	507 (59.9)	0	0	478 (26.8)	29 (5.8)
**Number of hemoglobin measurements over exposure assessment period**^**C**^						
Mean (SD)	5.5 (5.0)	5.8 (4.7)	5.9 (5.6)	4.2 (3.9)	5.5 (5.0)	5.4 (5.2)
Median (IQR)	4.0 (2.0, 7.0)	4.0 (2.0, 7.0)	4.0 (2.0, 7.0)	3.0 (2.0, 5.0)	4.0 (2.0, 7.0)	3.0 (2.0, 6.0)
**Hemoglobin measurements, PPPM**						
Mean (SD)	0.6 (0.6)	0.6 (0.5)	0.7 (0.6)	0.5 (0.4)	0.6 (0.6)	0.6 (0.6)
Median (IQR)	0.4 (0.2, 0.8)	0.4 (0.2, 0.8)	0.4 (0.2, 0.8)	0.3 (0.2, 0.6)	0.4 (0.2, 0.8)	0.3 (0.2, 0.7)
**Average time between hemoglobin measurements, months**^**d**^						
Mean (SD)	1.7 (1.6)	1.4 (1.4)	1.8 (1.6)	2.1 (1.7)	1.7 (1.6)	1.8 (1.6)
Median (IQR)	1.2 (0.6, 2.2)	0.9 (0.5, 1.7)	1.3 (0.7, 2.4)	1.6 (0.9, 2.8)	1.1 (0.6, 2.2)	1.3 (0.7, 2.4)
**Average time between hemoglobin measurements, *n (%)***^**d**^						
< 1 month	972 (42.7)	473 (55.9)	376 (37.5)	123 (28.7)	776 (43.5)	196 (39.4)
1–3 months	1,106 (48.5)	321 (37.9)	532 (53.0)	253 (59.0)	850 (47.7)	256 (51.5)
4–6 months	166 (7.3)	46 (5.4)	76 (7.6)	44 (10.3)	130 (7.3)	36 (7.2)
7–9 months	35 (1.5)	6 (0.7)	20 (2.0)	9 (2.1)	26 (1.5)	9 (1.8)

^a^Patients were classified as having a mean hemoglobin level below the target range (< 10.0 g/dL), within the target range (10.0‒11.5 g/dL), or above the target range (> 11.5 g/dL)

^b^Patients were classified as having either a high time-in-range (TiR; ≥ 60% of all hemoglobin measurements within the target range of 10.0‒11.5 g/dL) or a low TiR (< 60% of all hemoglobin measurements within the target range of 10.0‒11.5 g/dL)

^c^To ensure hemoglobin measurements were distinct events, measurements occurring within 7 days were considered as one measurement episode and the hemoglobin values were averaged

^d^Time between hemoglobin measurements was assessed as the average time between. consecutive hemoglobin measurements per patient and subsequently evaluated at the population-level

IQR, interquartile range; PPPM; per patient per month; SD, standard deviation; TiR, time-in-range

## All-cause HCRU and costs by mean hemoglobin level

### Within versus below the target range

**Fig. 3:**
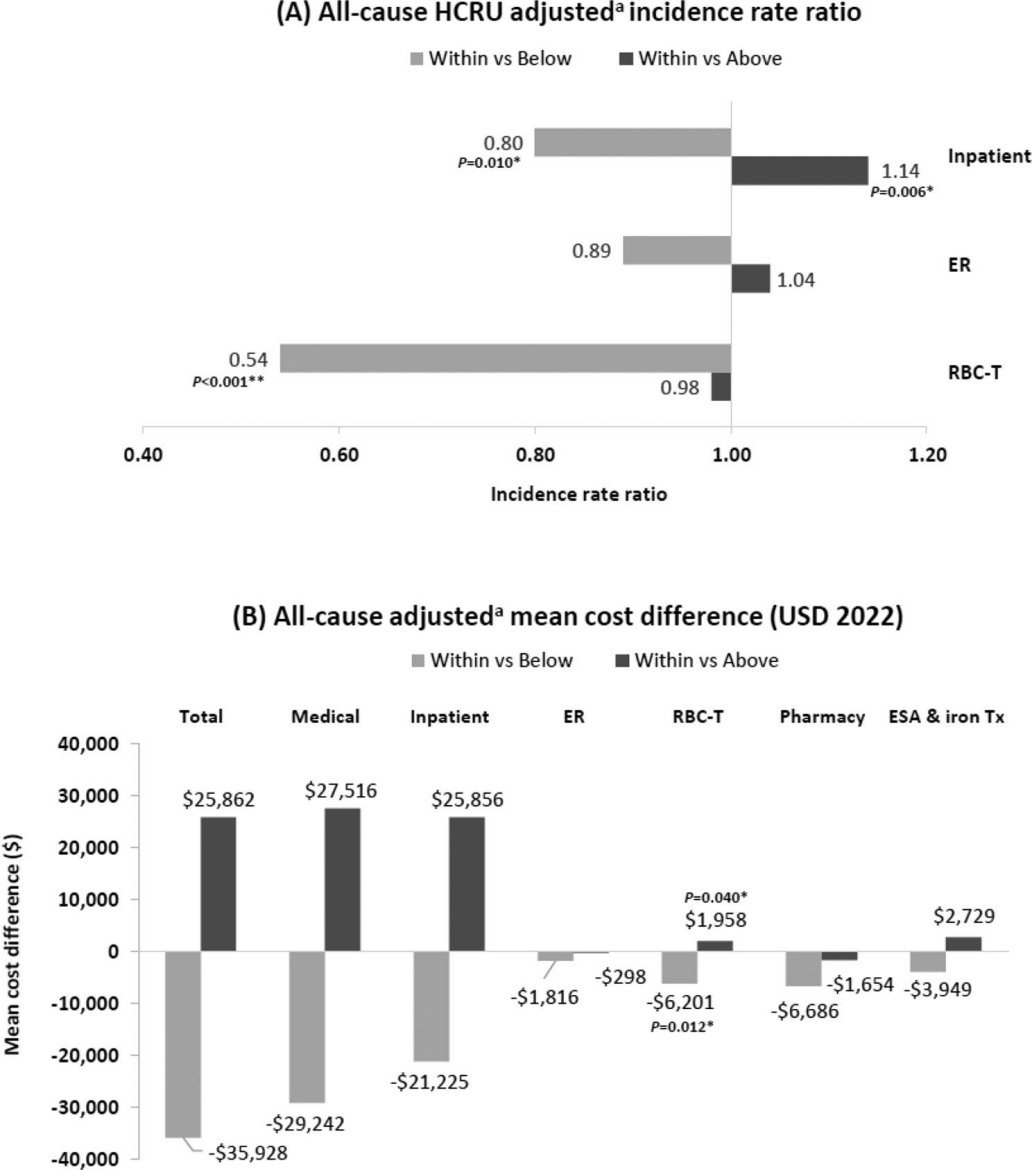
All-cause HCRU and mean costs: hemoglobin level within versus below and above the target range. *P-value < 0.05; **P-value < 0.001. Adjusted^a^ differences in (**A**) all-cause HCRU incidence rate ratio and (**B**) mean costs between groups of patients with hemoglobin level within versus below and within versus above the target range. ^a^Estimates were weighted based on the IPW approach and further adjusted for any covariates that were unbalanced after weighting (i.e., standardized difference > 10%), including ethnicity, RBC-T visits during the baseline period, and anemia of ESKD treatment on index during the baseline period. ER, emergency room; ESA, erythropoiesis-stimulating agents; ESKD, end-stage kidney disease; HCRU, healthcare resource utilization; IPW, inverse probability weighting; RBC-T, red blood cell transfusion; Tx, treatment; USD, United States dollars

Patients with a mean hemoglobin level within the target range had lower incidence rates of all-cause HCRU PPY than those with a mean hemoglobin level below the target range, particularly for inpatient admissions (1.48 versus 1.79) and RBC transfusions (0.45 versus 0.68) (Table 3). After adjustment, this reduction remained significant with a 46% lower rate of RBC transfusion (IRR, 0.54; 95% CI, 0.41–0.70; *p* < 0.001) and a 20% lower rate of inpatient visits (IRR, 0.80; 95% CI, 0.68–0.95; *p* = 0.010; Fig. 3A). Annual mean total healthcare costs were also lower (total healthcare costs: $120,168 versus $156,096), including mean RBC transfusion costs ($3,520 versus $9,721). After adjustment, total healthcare costs were numerically lower, although the difference did not attain statistical significance. The reduction in RBC transfusion costs remained significant with a mean cost reduction of $6,201 (95% CI, $11,968–$1,332; *p* = 0.012; Fig. 3B).

**Table 3:** All-cause HCRU and costs (PPY) during outcomes assessment period by study comparator groups (weighted^a^ sample)

	**Mean hemoglobin during the exposure assessment period**^**b**^			**TiR during the exposure assessment period**^**c**^	
	Below target range (N = 846)	Within target range (N = 1,004)	Above target range (N = 429)	Low TiR (N = 1,782)	High TiR (N = 497)
Total person years	1,446.6	1,880.2	820.9	3,220.7	948.3
**Outcomes assessment period, years**					
Mean (SD)	1.7 (1.3)	1.9 (1.3)	1.9 (1.3)	1.8 (1.3)	1.9 (1.3)
Median (IQR)	1 (1, 2)	2 (1, 3)	2 (1, 3)	2 (1, 3)	2 (1, 3)
**All-cause HCRU, PPY**					
Emergency room visits	1.9	1.9	1.6	1.9	1.8
Inpatient admissions	1.8	1.5	1.2	1.6	1.3
RBC transfusion^d^	0.7	0.5	0.3	0.5	0.3
**Healthcare costs**^**d**^**, mean (SE), PPPY**					
Total healthcare cost^e^	156,096 (11,710)	120,168 (13,892)	94,306 (8,618)	136,186 (9,522)	102,266 (9,202)
Medical cost	137,556 (10,185)	108,314 (13,814)	80,798 (7,589)	120,428 (8,987)	90,637 (8,881)
ER visits cost	10,211 (1,037)	8,395 (626)	8,692 (1,264)	9,655 (666)	7,447(739)
Inpatient admissions cost	117,624 (8,985)	96,399 (13,561)	70,544 (7,124)	105,099 (8,584)	78,636 (8,024)
RBC transfusion cost^d^	9,721 (2,167)	3,520 (721)	1,563 (460)	5,674 (1,054)	4,554 (1,368)
All-cause pharmacy cost	18,540 (5,490)	11,854 (1,351)	13,508 (2,988)	15,758 (2,934)	11,629 (1,915)
Anemia of ESKD-related treatment cost^f^	18,168 (5,563)	14,219 (1,879)	11,490 (2,764)	16,210 (3,039)	13,698 (2,302)

^a^Weights were derived from the inverse of the propensity score generated from multinomial regression models adjusting for potential confounding variables. The following variables were included in the propensity score models: age, sex, race, geographic region, medical insurance type, year of anemia ESKD treatment, type of anemia ESKD treatment over the exposure assessment period, dialysis treatment modalities, dialysis vintage, proportion of patients switching dialysis treatment modalities, other cardiovascular disorder, arteriosclerotic heart disease, chronic obstructive pulmonary disorder, Quan-Charlson comorbidity index, BMI, and all-cause HCRU

^b^Patients were classified as having a mean hemoglobin level below the target range (< 10.0 g/dL), within the target range (10.0‒11.5 g/dL), or above the target range (> 11.5 g/dL)

^c^Patients were classified as having either a high time-in-range (TiR; ≥ 60% of all hemoglobin measurements within the target range of 10.0‒11.5 g/dL) or a low TiR (< 60% of all hemoglobin measurements within the target range of 10.0‒11.5 g/dL)

^d^RBC transfusion visits were defined as any medical claim with a procedure or revenue code for a RBC transfusion. RBC transfusions occurred during outpatient visits (59.9%), inpatient visits (31.1%), other visits (8.8%), and ER visits (0.1%)

^e^Total healthcare cost included medical costs (inpatient, ER, and RBC transfusion costs) and pharmacy costs

^f^Anemia of ESKD-related treatment costs were defined as the medical and pharmacy costs associated with receipt of ESAs and supplemental iron. (IV or oral)

BMI, body mass index; ER, emergency room; ESA, erythropoiesis-stimulating agent; ESKD, end-stage kidney disease; HCRU; healthcare resource utilization; IQR, interquartile range; IV, intravenous; PPY, per person year; PPPY, per person per year; RBC, red blood cell; SD, standard deviation; TiR, time-in-range

Consistent with the analysis of the overall population, the newly and previously treated subgroup analysis found that a mean hemoglobin level within the target range was associated with a significantly lower incidence rate of RBC transfusion PPY (newly treated: 42% [IRR: 0.58; 95% CI: 0.35–0.95; *p* = 0.031]; previously treated: 54% [IRR: 0.46; 95% CI: 0.32–0.68; *p* < 0.001]) and inpatient visits (newly treated: 21% [IRR: 0.79; 95% CI: 0.64–0.98; *p* = 0.034]; previously treated: 23% [IRR: 0.77; 95% CI: 0.61–0.98; *p* = 0.031]), compared with a mean hemoglobin level below the target range (Supplementary Fig. 1). Accordingly, a mean hemoglobin level within the target range was associated with numerically lower annual costs for RBC transfusion in both newly and previously treated patients compared to patients with a mean hemoglobin level below the target range, although differences did not reach statistical significance (newly treated: mean cost difference: -$8,367; 95% CI, -$18,624–$553; *p* = 0.076; previously treated: -$5,029; 95% CI, -$30,315–$577; *p* = 0.088).

### Within versus above the target range

Patients with a mean hemoglobin level within the target range had higher incidence rates of all-cause HCRU PPY than those with a mean hemoglobin level above the target range, including ER visits (1.92 versus 1.59), inpatient admissions (1.48 versus 1.19), and RBC transfusions (0.45 versus 0.34) (Table 3). After adjustment, patients with a mean hemoglobin level within the target range versus above target range had a 14% significantly higher incidence rate of inpatient visits PPY (IRR, 1.14; 95% CI, 1.04–1.25; *p* = 0.006; Fig. 3A).

Annual mean total healthcare costs were also higher ($120,168 versus $94,306) but this difference was not statistically significant even after adjustment. RBC transfusion costs for patients with a mean hemoglobin within the target range were higher than for patients with a mean hemoglobin level above the target range (mean: $3,520 versus $1,563 PPPY), and the adjusted difference was significant (mean cost difference: $1,958; 95% CI, $133–$3,505; *p* = 0.040; Fig. 3B).

In the newly and previously treated subgroup analysis, previously treated patients with a mean hemoglobin level within the target range had statistically significant increases in RBC transfusions (62% [IRR: 1.62; 95% CI: 1.27–2.07; *p* < 0.001]) and inpatient visits (18% [IRR: 1.18; 95% CI: 1.03–1.35; *p* = 0.014]) versus patients with a mean hemoglobin level above the target range. Previously treated patients with a mean hemoglobin level within the target range also had significantly higher annual mean medical and inpatient admission costs compared to patients above the target range (Supplementary Fig. 1). There were no significant differences in HCRU or total medical costs between patients with a mean hemoglobin level within versus above the target range among newly treated patients except for anemia treatment costs (defined as ESAs and iron treatments) which were significantly increased in patients with a mean hemoglobin level within the target range compared to patients above (mean cost difference: $5,192; 95% CI, $1,729–$10,757; *p* = 0.012).

## All-cause HCRU and costs by TiR comparator groups

**Fig. 4:**
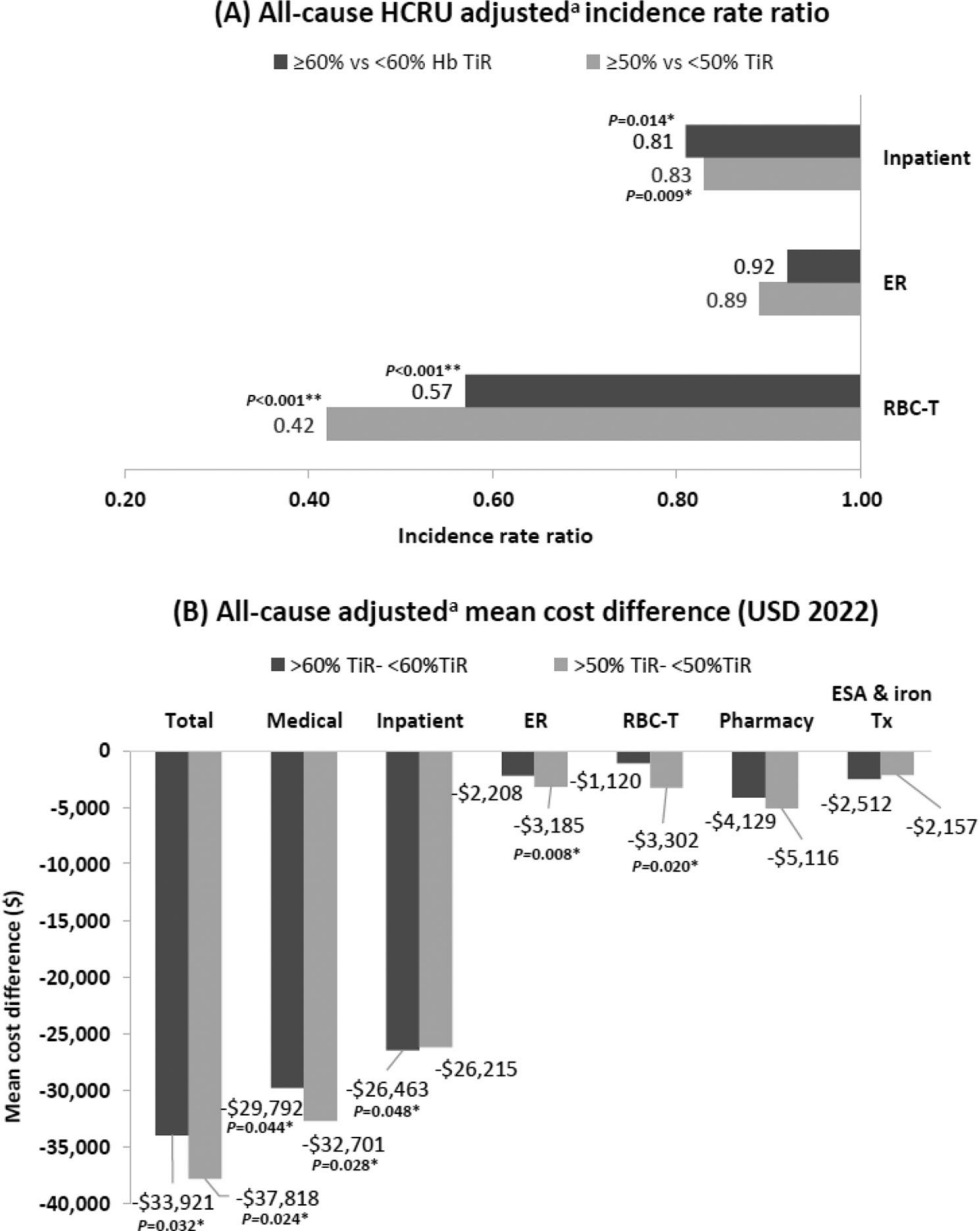
All-cause HCRU and mean costs between groups of patients with high TiR versus low TiR. *P-value < 0.05; **P-value < 0.001. Adjusted^a^ difference in (**A**) all-cause HCRU incidence rate ratio and (**B**) mean costs between groups of patients with high TiR versus low TiR. ^a^Estimates were weighted based on the IPW approach and further adjusted for any covariates that were unbalanced after weighting (i.e., standardized difference > 10%), including ethnicity, RBC-T visits during the baseline period, and anemia of ESKD treatment on index during the baseline period. ER, emergency room; ESA, erythropoiesis-stimulating agents; ESKD, end-stage kidney disease; HCRU, healthcare resource utilization; IPW, inverse probability weighting; RBC-T, red blood cell transfusion; TiR, time in range; Tx, treatment; USD, United States dollars

Patients in the high TiR group had lower incidence rates of all-cause HCRU PPY than patients in the low TiR group, including ER visits (1.78 versus 1.88), inpatient admissions (1.32 versus 1.63), and RBC transfusions (0.30 versus 0.54) (Table 3). After adjustment, these reductions remained significant with a 43 and 19% lower incidence rate of RBC transfusion visits (IRR, 0.57; 95% CI, 0.42–0.79; *p* < 0.001) and inpatient visits (IRR, 0.81; 95% CI, 0.69–0.96; *p* = 0.014) for the high TiR versus the low TiR group (Fig. 4A). Furthermore, patients in the high TiR group had lower annual total mean healthcare costs than patients in the low TiR group ($102,266 versus $136,186) after adjustment, including lower mean RBC transfusion costs ($4,554 versus $5,674), which was significant after adjustment (total mean healthcare cost difference: $33,921; *p* = 0.032; Fig. 4B). Similar trends were observed in sensitivity analyses where the threshold for high TiR was lowered from ≥ 60% to ≥ 50% of all hemoglobin measurements within the target range.

## Discussion

In this US database analysis of 2,279 patients with anemia of ESKD on dialysis, patients with a mean hemoglobin level within the target range (10.0–11.5 g/dL) had significantly lower incidence rates of inpatient visits and RBC transfusions PPY than patients with a mean hemoglobin level below the target range. Results were consistent in both newly and previously treated patients and were corroborated in patients with high TiR who used significantly fewer healthcare resources and incurred significantly lower healthcare costs per year than patients with low TiR. This finding aligns with published literature that investigated the impact of hemoglobin variability on hospitalization among patients with anemia of CKD on hemodialysis (*n* = 1,634) [22]. The study found that patients with a hemoglobin level below target range in terms of time, area, and number of excursions had more frequent hospitalization and increased length of stay than patients with a mean hemoglobin within the target range of 11.0–12.5 g/dL [22]. Together, these data highlight the value of maintaining hemoglobin levels within the recommended target range to reduce economic burden.

Our analysis found that patients with a mean hemoglobin level within the recommended target range had more inpatient visits, higher inpatient visit costs, and higher RBC transfusion costs per year than patients with a mean hemoglobin level above the target range. Prior studies noted benefits in HCRU and costs when treating patients’ hemoglobin level to targets above 12 g/dL [23, 24]. In a US retrospective, longitudinal 6-month study of patients (*N* = 44,550) with CKD on hemodialysis, those with a baseline hemoglobin level of ≥ 13 g/dL had fewer hospitalizations than patients with a baseline hemoglobin level of < 9 g/dL (1.65 versus 2.45 visits), although differences were not statistically significant after adjusting for covariates. Furthermore patients with a hemoglobin level > 12 g/dL did not have an increase in hospitalizations or length of stay [24]. A prospective study conducted across Spain showed that treatment of patients (*n* = 115) on hemodialysis to a hemoglobin target of 12.5 g/dL after 6 months resulted in a 58% reduction in hospitalizations and a 69% reduction in length of hospital stay compared to a mean baseline hemoglobin level of 10.2 g/dL [23]. Handleman et al. found that patients with a hemoglobin level above target range in terms of time and number of excursions had a reduced frequency of hospitalization and no increase in length of stay than patients with a mean hemoglobin within the target range of 11.0–12.5 g/dL [22].

While these studies together with our current findings may suggest possible reduction in HCRU and costs for patients with mean hemoglobin level above versus within target range, the following differences should be noted. First, the mean hemoglobin level for the above the target range group was 12.3 g/dL and 75% of patients had a value ≤ 12.6 g/dL, in contrast to the aforementioned studies that reported higher hemoglobin levels. The relatively lower mean hemoglobin of the above the target range group in the current study is likely reflective of the conservative treatment approach in concordance with current guidelines, whereas the other studies pre-dated pivotal trials demonstrating increased cardiovascular risk and mortality treating to higher hemoglobin targets [12–16]. Secondly, while we noted reductions in inpatient visits and transfusions for those above versus within target range, cost reductions were not statistically significant and do not suggest that patients would have lower costs if treated to higher hemoglobin targets. It is possible that the modest sample sizes of the stratification groups may have impacted the ability to detect statistical significance in cost differences. A future study with greater power may be needed to confirm the impact on cost for patients treated to higher hemoglobin targets.

Cardiovascular event rates or death rates during follow up were not measured in our study, limiting clinical benefit-risk assessment for the known cardiovascular risks of higher hemoglobin levels above 11.5 g/dL, and which could potentially bias results towards those with better health and/or loss from early death for patients with a mean hemoglobin level above the target range. The increased risk of major adverse cardiovascular events observed when treating patients to higher hemoglobin targets based on published literature may advocate for maintaining patients within the target range [13, 15, 28], given that our study did not find significant differences in HCRU and costs for the above versus within the target range groups. KDIGO 2012 acknowledges that some patients may benefit from hemoglobin levels above 11.5 g/dL to improve quality of life [3], although updated KDIGO guidelines are anticipated in 2025.

Future studies investigating personalized target hemoglobin levels among patients with lower cardiovascular risks are needed to ascertain the clinical and economic impact of treating patients with ESKD on dialysis to a higher hemoglobin target range. A consensus document from the European Anaemia of CKD Alliance encourages a shift to a personalized approach to treating anemia of CKD, involving shared decision-making with patients based on individual needs, such as comorbidities, activity level, and quality of life expectation [29]. Technological advancements in predicting hemoglobin levels may help determine future hemoglobin levels in patients on hemodialysis and recommend an individualized dose of ESA treatment, which could potentially prevent high HCRU if their anemia is better controlled [30].

A strength of this study is the use of a large claims and EHR database and two distinct definitions of hemoglobin stability, providing a robust assessment of the economic impact of hemoglobin stability. However, this study has limitations that must be considered when interpreting the results. First, bundled reimbursement from US dialysis organizations may result in billing claims that do not reflect all healthcare services (e.g., laboratory tests, anemia of ESKD medication). Therefore, some hemoglobin measures may have not been captured, resulting in misclassification of patients into our pre-defined comparator groups. Second, patients were required to have ≥ 9 months of EHR activity following the anemia of ESKD treatment date which may have introduced selection bias. Third, timing of hemoglobin measurements may not have been uniform across the exposure assessment period. Hemoglobin may have been disproportionately monitored during periods when levels were below or above the target range, leading to potential misclassification of patients stratified by TiR. Lastly, the de-identified Optum^®^ Market Clarity data does not include Medicare fee-for-service patients, who represent approximately half of patients who are dialysis-dependent in the US [31], therefore, our results may have limited generalizability to patients with anemia of ESKD on chronic dialysis covered by Medicare fee-for-service. This study also did not explore direct out-of-pocket and indirect costs, therefore the results may underestimate the full economic burden for patients with anemia of ESKD on chronic dialysis. Future work is needed to determine the direct and indirect economic burden to patients.

## Conclusions

Hemoglobin levels within the target range of 10.0–11.5 g/dL, and having a higher percentage of TiR, were associated with positive impacts on RBC transfusion frequency, HCRU, and healthcare costs among dialysis-dependent patients with anemia of ESKD. Future research is warranted to understand why patients with hemoglobin levels within the target range had a higher rate of inpatient visits than those with hemoglobin levels above the target range. Further work is also needed to shed light on potential HCRU and economic benefits that might be associated with a higher hemoglobin target range, as seen in our study.

## Electronic supplementary material

Below is the link to the electronic supplementary material.

Supplementary Material 1

## Data Availability

The Optum’s de-identified Market Clarity Data (Optum^®^ Market Clarity) database that support the findings of this study was used under license and therefore, are not publicly available. For inquiries on the dataset analyzed in this study, please contact Optum® (https://www.optum.com).

## Abbreviations

BMI: Body mass index
CKD: Chronic kidney disease
CI: Confidence intervals
ER: Emergency room
ESA: Erythropoiesis-stimulating agents
ESKD: End-stage kidney disease
HCRU: Healthcare resource utilization
IPW: Inverse probability weighting
IQR: Interquartile range
IRRs: Incidence rate ratios
IV: Intravenous
PPPM: Per patient per month
PPY: Per person year
PPPY: Per person per year
RBC: Red blood cell
RBC-T: Red blood cell transfusion
SD: Standard deviation
TiR: Time-in-range
Tx: Treatment
US: United States
USD: United States dollars
