# Supplementary material for: Hemoglobin stability impact on healthcare resource utilization and costs among dialysis-dependent patients with anemia of end-stage kidney disease
Source: BMC Nephrol. 2025 Aug 18;26:466. doi: 10.1186/s12882-025-04390-y (PMC12363002; doi:10.1186/s12882-025-04390-y)
Supplement: Supplementary file 1 — Supplementary Material 1 [file 12882_2025_4390_MOESM1_ESM.docx]

**SUPPLEMENTARY MATERIAL**

**Hemoglobin stability impact on healthcare resource utilization and costs among dialysis-dependent patients with anemia of end-stage kidney disease**

**Table of contents**

| **Supplementary Table 1.** Unweighted patient characteristics by study comparator groups. | **Page 2** |
| --- | --- |
| **Supplementary Fig. 1.** Adjusted^a^ difference in incidence rate ratio of all-cause HCRU and costs between groups of patients with hemoglobin levels within versus below and within versus above the target range stratified by (A) newly and (B) previously treated with anemia of CKD treatment. | **Page 5** |

**Supplementary results**

**Supplementary Table 1.** Unweighted patient characteristics by study comparator groups.

|  | **Mean hemoglobin during the exposure assessment period^a^** | | | | **TiR during the exposure assessment period ^b^** | | |
| --- | --- | --- | --- | --- | --- | --- | --- |
| **Characteristic** | **Below target range (*N*=846)** | **Within target range (*N*=1,004)** | **Above target range  (*N*=429)** | **Std. diff**  **(%)** | **Low TiR**  **(*N*=1,782)** | **High TiR (*N*=497)** | **Std. diff**  **(%)** |
| **Age in years, mean (SD)** | 60.8 ± 13.4 | 61.8 ± 13.3 | 61.7 ± 12.6 | 7.39 | 61.3 ± 13.3 | 62.0 ± 13.1 | 5.88 |
| **Age in years, median (IQR)** | 62 (53,71) | 63 (53,72) | 62 (54,71) | ─ | 62 (54,71) | 64 (53,72) | ─ |
| **Male, *n* (%)** | 437 (51.7) | 523 (52.1) | 266 (62.0) | 0.87 | 962 (54.0) | 264 (53.1) | -1.74 |
| **Race, *n* (%)** | | | | | | | |
| White | 441 (52.1) | 477 (47.5) | 228 (53.1) | -9.25 | 898 (50.4) | 248 (49.9) | -0.99 |
| African American/Black | 316 (37.4) | 395 (39.3) | 147 (34.3) | 4.09 | 674 (37.8) | 184 (37.0) | -1.65 |
| Asian | 8 (0.9) | 28 (2.8) | 12 (2.8) | **13.65†** | 31 (1.7) | 17 (3.4) | **10.62†** |
| Other/unknown | 81 (9.6) | 104 (10.4) | 42 (9.8) | 2.62 | 179 (10.0) | 48 (9.7) | -1.30 |
| **Ethnicity, *n* (%)** | | | | | | | |
| Not Hispanic/Latino | 708 (83.7) | 796 (79.3) | 340 (79.3) | **-11.36†** | 1,462 (82.0) | 382 (76.9) | **-12.85†** |
| Hispanic/Latino | 65 (7.7) | 119 (11.9) | 47 (11.0) | **14.08†** | 168 (9.4) | 63 (12.7) | **10.37†** |
| Unknown | 73 (8.6) | 89 (8.9) | 42 (9.8) | 0.83 | 152 (8.5) | 52 (10.5) | 6.60 |
| **Medical insurance type, *n* (%)** | | | | | | | |
| Medicare | 415 (49.1) | 523 (52.1) | 202 (47.1) | 6.08 | 871 (48.9) | 269 (54.1) | **10.51†** |
| Commercial | 227 (26.8) | 275 (27.4) | 123 (28.7) | 1.26 | 507 (28.5) | 118 (23.7) | **-10.74†** |
| Medicaid | 138 (16.3) | 135 (13.4) | 68 (15.9) | -8.06 | 274 (15.4) | 67 (13.5) | -5.40 |
| Unknown | 66 (7.8) | 71 (7.1) | 36 (8.4) | -2.78 | 130 (7.3) | 43 (8.7) | 5.01 |
| **Anemia of ESKD treatment over the exposure assessment period, *n* (%)** | | | | | | | |
| ESA only | 111 (13.1) | 94 (9.4) | 33 (7.7) | **-11.92†** | 189 (10.6) | 49 (9.9) | -2.46 |
| Supplemental iron only | 65 (7.7) | 89 (8.9) | 105 (24.5) | 4.29 | 198 (11.1) | 61 (12.3) | 3.62 |
| Both | 670 (79.2) | 821 (81.8) | 291 (67.8) | 6.50 | 1,395 (78.3) | 387 (77.9) | -1.00 |
| **Dialysis treatment modalities, *n* (%)** | | | | | | | |
| Hemodialysis | 681 (80.5) | 870 (86.7) | 388 (90.4) | **16.68†** | 1,517 (85.1) | 422 (84.9) | -0.62 |
| Peritoneal dialysis | 130 (15.4) | 108 (10.8) | 56 (13.1) | **-13.71†** | 236 (13.2) | 58 (11.7) | -4.77 |
| Unknown | 111 (13.1) | 94 (9.4) | 24 (5.6) | **-11.92†** | 178 (10.0) | 51 (10.3) | 0.90 |
| **Dialysis vintage, *n* (%)** | | | | | | | |
| 1–4 months | 295 (34.9) | 333 (33.2) | 141 (32.9) | -3.59 | 646 (36.3) | 123 (24.7) | -**25.18†** |
| >4 months | 551 (65.1) | 671 (66.8) | 288 (67.1) | ─ | 1,136 (63.7) | 374 (75.3) | ─ |
| **Dialysis vintage, months** | | | | | | | |
| Mean (SD) | 7.3 (5.1) | 7.4 (4.9) | 7.5 (4.9) | 3.74 | 7.1 (5.0) | 8.3 (4.6) | **24.46†** |
| Median (IQR) | 10 (1, 12) | 10 (1, 12) | 10 (2, 12) | ─ | 10 (1, 12) | 11 (4, 12) | ─ |
| **ESKD-related comorbidities, *n* (%)** | | | | | | | |
| Hypertension | 808 (95.5) | 954 (95.0) | 413 (96.3) | -2.30 | 1,708 (95.8) | 467 (94.0) | -8.57 |
| Diabetes | 626 (74.0) | 715 (71.2) | 305 (71.1) | -6.24 | 1,304 (73.2) | 342 (68.8) | -9.63 |
| Hyperlipidemia | 574 (67.8) | 708 (70.5) | 306 (71.3) | 5.78 | 1,238 (69.5) | 350 (70.4) | 2.07 |
| Heart failure | 496 (58.6) | 552 (55.0) | 230 (53.6) | -7.37 | 1,008 (56.6) | 270 (54.3) | -4.51 |

†Standardized difference >10% indicative of a meaningful imbalance between groups.
^a^Patients were classified as having a mean hemoglobin level below the target range (<10.0 g/dL), within the target range (10.0‒11.5 g/dL), or above the target range (>11.5 g/dL).
^b^Patients were classified as having either a high time-in-range (TiR; ≥60% of all hemoglobin measurements within the target range of 10.0‒11.5 g/dL) or a low TiR (<60% of all hemoglobin measurements within the target range of 10.0‒11.5 g/dL).

ESA, erythropoiesis-stimulating agent; ESKD, end-stage kidney disease; IQR, interquartile range; SD, standard deviation; Std. diff., standardized difference; TiR, time-in-range.

**Supplementary Fig. 1.** Adjusted^a^ difference in incidence rate ratio of all-cause HCRU and costs between groups of patients with hemoglobin levels within versus below and within versus above the target range stratified by (A) newly and (B) previously treated with anemia of CKD treatment.


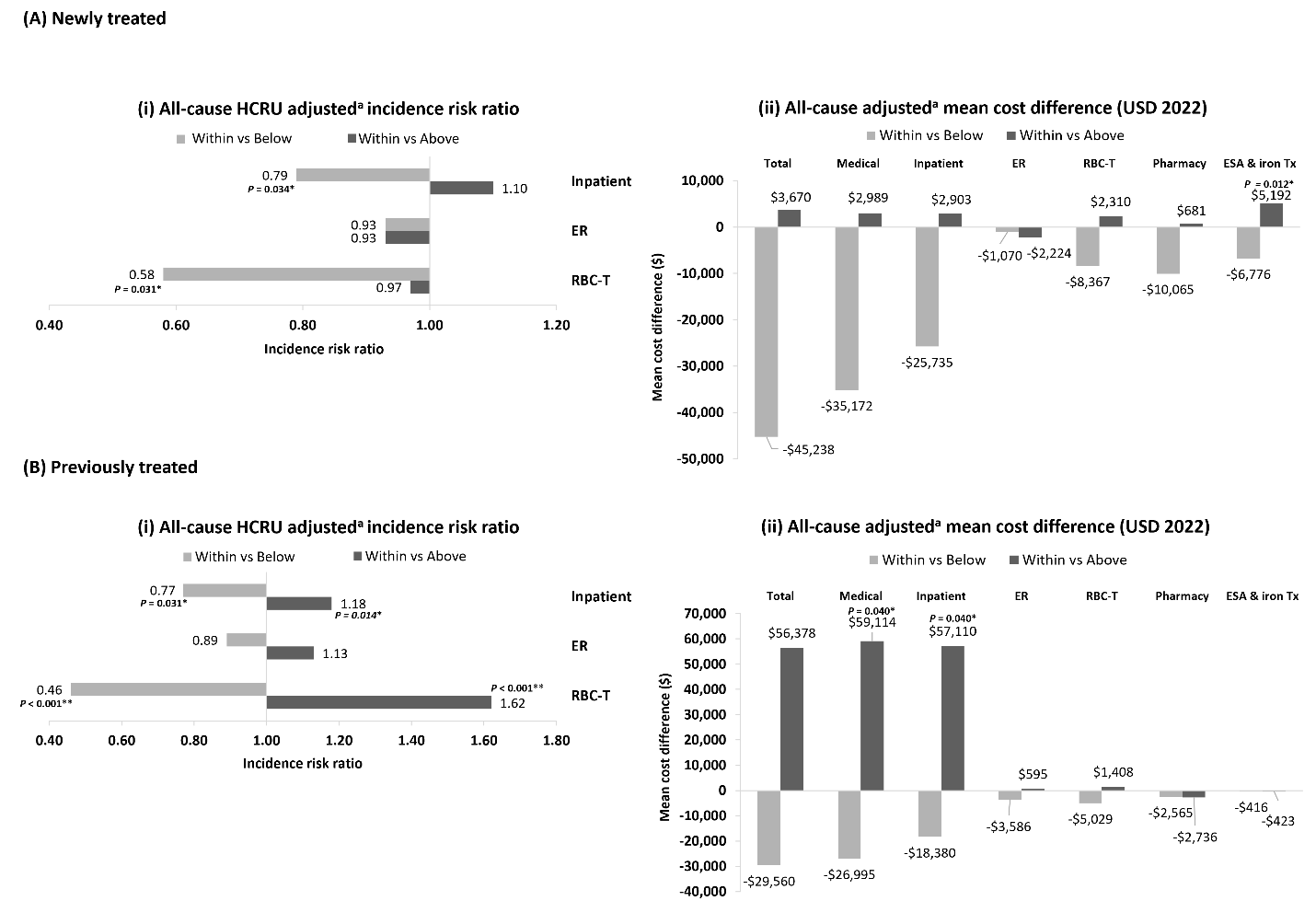
**P*-value<0.05; ***P*-value<0.001.

^a^Estimates were weighted based on the IPW approach and further adjusted for any covariates that were unbalanced after weighting (*i.e.,* standardized difference >10%), including ethnicity, RBC-T visits during the baseline period, and anemia of ESKD treatment on index during the baseline period.

ER, emergency room; ESA, erythropoiesis-stimulating agents; ESKD, end-stage kidney disease; HCRU, healthcare resource utilization; IPW, Inverse Probability Weighting; RBC-T, red blood cell transfusion;
Tx, treatment; USD, US dollars.
